# Gut microbiota alterations in critically ill older patients: a multicenter study

**DOI:** 10.1186/s12877-022-02981-0

**Published:** 2022-04-28

**Authors:** Mesa Victoria, Valdés-Duque Beatriz Elena, Giraldo-Giraldo Nubia Amparo, Jailler-R Ana María, Giraldo-Villa Adriana, Acevedo-Castaño Irene, Yepes-M Mónica Alejandra, Barbosa-Barbosa Janeth, Agudelo-Ochoa Gloria María

**Affiliations:** 1 Food and Human Nutrition Research Group, Universidad de Antioquia (UdeA), Medellín, Antioquia Colombia; 2 INSERM UMR S-1139 3PHM, Université Paris Cité, 75006 Paris, France; 3 Biosciences Research Group, Institución Universitaria Colegio Mayor de Antioquia, Medellín, Antioquia Colombia; 4 Department of Nutrition, Hospital San Vicente Fundación, Rionegro, Antioquia Colombia; 5 Department of Nutrition, Hospital Pablo Tobón Uribe, Medellín, Antioquia Colombia; 6 Department of Nutrition, Hospital General de Medellín, Medellín, Antioquia Colombia; 7 Department of Nutrition, Hospital San Vicente Fundación, Medellín, Antioquia Colombia; 8 Department of Nutrition, Clínica Las Américas, Medellín, Antioquia Colombia

**Keywords:** Older adults, Critically ill, Gut microbiota, ICU, Sepsis, Dysbiosis

## Abstract

**Background:**

Aging generates changes in the gut microbiota, affecting its functionality. Little is known about gut microbiota in critically ill older adults. The objective of this study was to describe the profile of gut microbiota in a cohort of critically ill older adults.

**Methods:**

This observational study was conducted in five health institutions. Over a 6-month study period, critically ill patients over 18 years old who were admitted to the intensive care unit were enrolled. Fecal microbiota profiles were determined from 155 individuals, over 60 years old (*n* = 72) and under 60 years old (*n* = 83). Gut microbiota was analyzed by sequencing the V3-V4 region of the 16S rRNA gene. Alpha and beta diversity, operational taxonomic units and the interaction of gut microbiota with variables under study were analyzed. Amplicon sequence variants (ASVs) specifically associated with age were recovered by including gender, discharge condition, BMI, ICU stay and antibiotics as covariates in a linear mixed model.

**Results:**

In older adults, sepsis, malnutrition, antibiotic prescription and severity (APACHE and SOFA scores) were higher than in the group under 60 years of age. Alpha diversity showed lower gut microbiota diversity in those over 60 years of age (*p* < 0.05); beta diversity evidenced significant differences between the groups (PERMANOVA = 1.19, *p =* 0.038). The microbiota of the adults under 60 years old showed greater abundance of *Murdochiella*, *Megasphaera*, *Peptoniphilus* and *Ezakiella*, whereas those over 60 years old *Escherichia*-*Shigella* and *Hungatella* were more abundant.

**Conclusion:**

The gut microbial community was altered by different factors; however, age significantly explained the variability in critically ill patients. A lower presence of beneficial genera and a higher abundance of pathogens was observed in adults over 60 years old.

**Supplementary Information:**

The online version contains supplementary material available at 10.1186/s12877-022-02981-0.

## Introduction

The intestine hosts a group of living microorganisms called microbiota [1], which coexists in a dynamic equilibrium with the host, and which, under normal conditions, has metabolic and structural functions, in addition to regulating immunity and systemic inflammation [2, 3]. In critical illness, when the organism suffers a physical (trauma, surgery, burn) or biological (infection) aggression, the intestine is considered the motor of infectious complications and multiorgan dysfunction syndrome (MODS), both associated with alterations of the intestinal epithelium, the immune function and the endogenous microbiota [4]. During critical illness, the conditions of the epithelium and the mucosal barrier change and alterations in the usual equilibrium of the gut microbiota occur, both in the abundance of bacteria and in their diversity, a condition called intestinal dysbiosis, in which the dominance of a certain taxon is frequent [1]. Compared to healthy individuals, in the critically ill patients the pathogenic bacteria, such as *Pseudomonas aeruginosa*, *Escherichia/Shigella, Enterococcus* or *Staphylococcus,* have the ability to compete more easily with other members of the gut microbiota, favoring changes in their composition [5]. It has been suggested that dysbiosis is a consequence, in addition to alterations in the intestinal mucosa layer, of changes in the host’s response to physiological stress, and to the medical care that the patient receives in the ICU (intensive care unit), especially the use of antibiotics, the feeding route, invasive procedures that can alter the natural barrier mechanisms, and the effect of other drugs such as vasopressors, opioids and mucosal protectors [6, 7].

It has been proposed that the composition and diversity of gut microbiota in healthy individuals changes with age, although between the third and seventh decade of life it remains stable, it may still present a slow deterioration in functionality. The variability of gut microbiota is attributed to biological, geographical and environmental factors. In older adults, diet, chronic diseases and the frequent use of medications, especially antibiotics, may have stronger influence [8].

In general, significant alterations in the phylogenetic diversity of gut microbiota have been reported in critically ill patients after their admission to the ICU. Compared to healthy volunteers, a decrease in the total of obligate anaerobes and an increase in pathogenic bacteria have been found in these patients with age range from 16 to 81 years old, a follow-up associated with the presence of complications such as sepsis and severe systemic inflammatory response syndrome (SIRS) reflected in increased mortality [3, 9]. In response to the release of opioids, severe modifications in the gut microbiota structure have been described during the process of a prolonged critical illness, finding that up to 30% of the patients studied presented a low diversity of microorganisms (1–4 bacterial taxa) compared to approximately ~40 communities identified in healthy individuals, which in turn were highly pathogenic and resistant to multiple drugs [10]. In another study, a significant change in the fecal bacterial composition was observed in critically ill patients with mean age of 64 years with and without sepsis in comparison with controls (healthy subjects), reporting the presence of only one bacterial genus in more than 50% of them, in addition to extreme interindividual differences [11].

Over the last decade, interest in gut microbiota has grown as an important component of the pathophysiology of a large number of conditions that affect critically ill patients, including sepsis [12, 13]. However, on the profile of gut microbiota in the critically ill older patient, little information is available. In most countries, this population group is increasing, therefore it is of interest to know the profile of its gut microbiota during critical illness, and in the future, to propose strategies for its modulation [3, 14]. Our aim in the present study was to describe the gut microbiota profile in a cohort of critically ill older patients and compare them with young adults (under 60 years of age). We also evaluated the impact of age on microbiota findings in critical care patients**.**

## Materials and methods

### Study design

This observational and multicenter study was conducted in the ICUs from third and ﻿fourth-level hospitals of five highly-complexity health institutions in the cities of Medellín and Rionegro (Colombia).

### Subject recruitments

The study population consisted of patients hospitalized in the ICUs of the five health institutions (Hospital Pablo Tobón Uribe, Hospital San Vicente Fundación Medellín, Hospital San Vicente Fundación Rionegro, Clínica las Américas and Hospital General de Medellín). The sample was made up of critically ill adult patients over 18 years old who were admitted to the ICU during a period of 6 months and met the inclusion criteria.

### Inclusion and exclusion criteria

Women and men over than 18 years old admitted to the ICU were enrolled. Patients in terminal condition, with colostomy or ileostomy, pregnant or lactating women and homeless people were excluded.

### Study protocol

The study was defined from the admission of the patient to the ICU (once the inclusion criteria were met and before starting nutritional support, a stool sample was obtained to evaluate gut microbiota) until discharge from the ICU (record on days of hospital stay and condition of discharge from the ICU). Information was collected on the variables of interest, and until discharge from the ICU, the complications presented by the patient were recorded. The following data were obtained from each patient’s clinical history: demographic (age and sex), clinical (clinical diagnosis upon admission to ICU, Apache II, SOFA, antibiotics, diagnosis of sepsis according to the criteria defined by the Third Consensus definition of sepsis [15]), anthropometric (BMI), biochemical (PCR, blood glucose); the hospital stay was measured in days from the patient’s admission to ICU until discharge; the appearance of a condition that occurred during hospitalization and that was independent of the cause of admission to ICU was considered a complication; the discharge condition referred to the state in which the patient left ICU, alive or dead.

﻿The patients or their relatives were personally informed of the study with the presence of the responsible clinician and signed informed consent forms. To coordinate the field work, an informative meeting was held in each hospital institution with nutrition, nursing and clinical laboratory personnel to present the study. All the personnel participating in the collection and processing of the information received training and standardization in everything related to the techniques and methodologies to be used to collect the samples and information.

### Fecal sampling

The stool sample for gut microbiota analysis was obtained by the nursing staff by means of rectal swabbing, using sterile polystyrene + viscose swabs, free of RNAse, DNAse and human DNA, in polypropylene tubes. In less than 15 min from collection, the samples were stored in the laboratories of each institution at −20 °C for subsequent transport and storage at −80 °C until the moment of analysis. To determine the abundance and diversity of gut microbiota, total genomic DNA was extracted from the patients’ stool samples, using commercial column kits (QIamp® DNA Stool Mini Kit-QIAGEN).

### DNA extraction, quantification and sequencing of barcoded amplicons on the Illumina MiSeq platform

The V3-V4 hypervariable regions of the 16S ribosomal ribonucleic acid (rRNA) gene were amplified using 1 μL of fecal DNA (25 ng on average). The polymerase chain reaction was performed within the following reaction conditions: 98 °C for 3 min; 27 cycles of 95 °C for 20 s, 55 °C for 20 s and 72 °C for 20 s. The primers contained the barcodes SD-Bact-0341-bS-17 (TAGCCTACGGGNGGCWGCAG) and SD-Bact-0785-aA-21 (ACTGACTACHVGGGTATCTAATCC) and each sample contained a unique 6 bp barcode. The barcoded PCR products were purified from triplicate reactions with an agarose gel band purification kit (illustra GFX PCR DNA and Gel Band Purification Kit, GE Healthcare, UK). Equimolar concentrations of PCR amplicons were quantified by fluorometric methods (Qubit 3.0 - Thermo Fisher Scientific, Waltham, MA, USA). The purified amplicons were pooled in equimolar amounts (~ 50 ng per sample) for library preparation. Sequencing was performed using an Illumina MiSeq paired-end system (2 x 300 bp) (Eurofins Genomics GmbH, Germany) following the manufacturer’s instructions.

### Data processing

The bioinformatic analysis of sequences was performed in the QIIME2 program (Quantitative Insights Into Microbial Ecology) version 2019.7. The samples were de-multiplexed, thus eliminating the associated primer and barcode sequences. The DADA2 method was implemented to detect and correct sequencing noises, remove chimeric sequences. The sequences were grouped in amplicon sequence variants (ASV) with 99% similarity. To classify the sequences according to their taxonomic information, the q2-feature-classifier plugin was used based on vsearch alignment method [16] with the SILVA v132 database [17].

### Statistical analysis

The study population was described by demographic and clinical variables, using measures of central tendency and dispersion for quantitative variables, qualitative variables were described by frequencies and percentages. The statistical analysis started with the evaluation of the normality of the continuous variables using the Kolmogorov-Smirnov test, if the normality was not met, ﻿non-parametric statistics were used. Two study groups were established according to age, older and younger than 60 years old. To determine the differences in the study groups by demographic and clinical variables, the Chi-2 test was used for qualitative variables; for quantitative variables the Student’s t test, or Mann-Whitney when appropriate, were used.

### Analysis of microbiota data

For the analysis of the diversity and richness of the gut microbiota, alpha diversity metrics were calculated to compare the diversity of the bacterial community within samples, including Chao1 richness, Shannon indices, Simpson’s inverse, and phylogenetic distance (PD); comparisons between groups were made using the ﻿non- parametric Wilcoxon rank sum test. Beta diversity, to identify the community structure between the two-age group and other variables of interest, was performed using a principal coordinate analysis (PCoA), calculating the weighted and unweighted UniFrac distances by permutation-based methods (PERMANOVA) (Adonis R package). Differential diversity and abundance analyzes were performed using the Phyloseq and Microbiome [18, 19] packages of RStudio v1.2.1335 [20] software. Through a mixed linear model (LMM), the association of the most abundant ASVs transformed logarithmically with the individual variables registered in the metadata were evaluated using the RStudio’s lme4 and nlme packages [21, 22]. Later, with the ASVs that showed a significant association (*p* < 0.05), the model was adjusted to control the effects of other variables, so that the resulting variation explained was independent of other variables and not subject to confusion by the correlated variables; each variable was the fixed factor and the others entered as covariates.

All graphs were made with the ggplot2 package from RStudio [23]. The general statistical analyzes were carried out with the R program [20]; in all cases, a *p* < 0.05 value was considered significant.

## Results

### Descriptive statistics of the study population

**Fig. 1:**
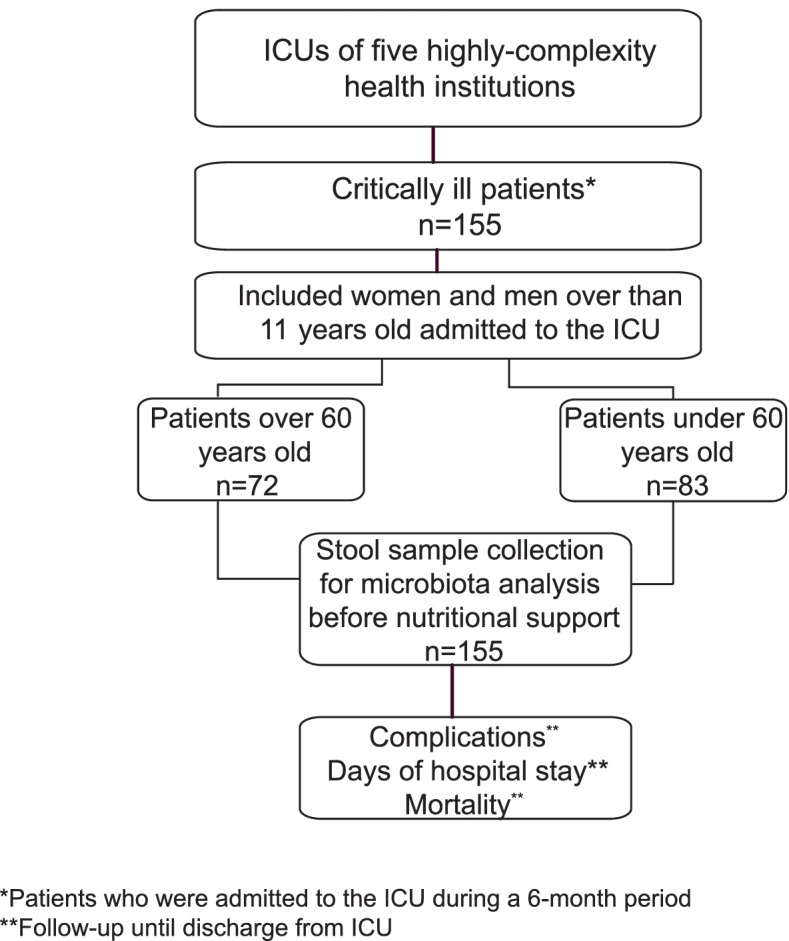
Flowchart of patients enrolled in the study

The final sample of the study consisted of 155 adults in critical illness, 72 adults over 60 years of age and as a control group, 83 adults under 60 years (Fig. 1).

The average age for the adults over 60 years was 73 ± 7 years and 42 ± 13 years for the adults under 60 years. In older adults, malnutrition (BMI classification), the presence of sepsis, SOFA and APACHE prognostic indexes, and the supply of antibiotics were significantly higher compared to the group under 60 years of age (*p* < 0.05). The general and clinical characteristics of the study population are described in Table 1 (Additional file 1: Table S1).

**Table 1:** General and clinical characteristics of the study population

Variable	Adults > 60 years ***n*** = 72	Adults < 60 years ***n*** = 83	***p***-value^**e**^
**Gender** ^a^			
Male	34 (47.2)	49 (59)	0.141
Female	38 (52.8)	34 (41)	
**BMI (Kg/m**^**2**^**)** ^b^	25.2 ± 4.1	24.4 ± 3.9	0.265
**BMI Classification** ^a^			
Under weight	20 (24.8)	5 (6)	0.001
Adequate	32 (44.4)	44 (53.0)	
Excess weight	20 (27.8)	34 (41.0)	
**Admission diagnosis** ^a^			
Trauma	4 (5.6)	22 (26.5)	0.817
Pulmonary	16 (22.2)	7 (8.4)	
Cardio cerebral vascular	14 (19.4)	10 (12)	
Renal/liver/pancreatic	6 (8.3)	4 (4.8)	
Surgery	4 (5,6)	5 (6,0)	
Infection disease	26 (36,1)	24 (28.9)	
Other diagnoses ^d^	2 (2.8)	11 (13.3)	
**Sepsis** ^a^			
Yes	42 (58.3)	30 (36.1)	0.006
No	30 (41.7)	53 (63.9)	
**Serum glucose (mg/dL)** ^c^	157 (124.2;188.5)	153 (121;206.3)	0.896
**CRP (mg/dL)** ^c^	8,9 (4.4;22.6)	11.9 (6.3;22.3)	0.437
**SOFA score** ^c^	3 (1;8)	1 (1;6)	0.029
**APACHE II score** ^b^	21 ± 8	15 ± 8	0.000
**Antibiotics** ^a^			
Yes	42 (58.3)	29 (34.9)	0.004
No	30 (41.7)	54 (65.1)	
**Complications** ^a^			
Yes	64 (88,9)	64 (77.1)	0.054
No	8 (11.1)	19 (22.9)	
**ICU stay (days)**	9 (5;15)	7 (4;17)	0.392
**Discharge condition** ^a^			0.206
Alive	49 (68.1)	64 (77.1)	
Dead	23 (31.9)	19 (22.9)	

^a^ Data are presented as mean and percentage

^b^ Data are presented as mean ± SD

^c^ Data are presented as median and interquartile ranges

^d^ Addictions, anxiety attacks, poisoning, circulatory disorder, neuromuscular disorder, cancer

^e^ Group-wise comparisons of the variables. For the qualitative variables the Chi-square test was used; according to the distribution of quantitative variables, the T-test or Mann-Whitney U test was applied for the independent groups; *p* < 0.05

*BMI* Body Mass Index

*CRP* C-reactive protein

*SOFA* Sequential Organ Failure Assessment

*APACHE II* Acute Physiology and Chronic Health Evaluation

*ICU* Intensive Care Unit

### Microbiota characterization

The data were imported into QIIME2 using the Casava1.8 paired ends protocol. A total of 3,268,240 sequences were obtained after filtering and checking chimera sequences of the 16S rRNA region V3-V4 from the 155 samples. The sequencing depth of the data processed by DADA2 ranged from 6074 to 46,988 sequences per sample.

**Fig. 2:**
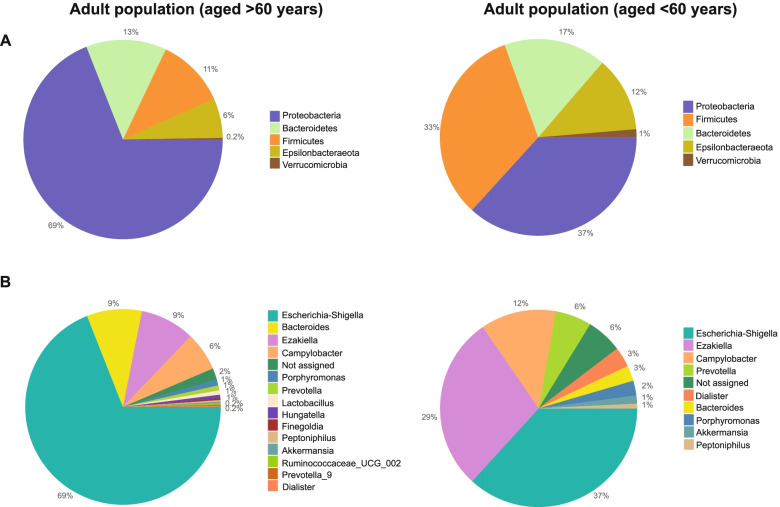
Representation of bacterial phyla (**A**) and genera (**B**) in the age groups

The taxonomic classification at the phylum level revealed that, in both age groups, the bacterial phyla detected were mainly Firmicutes, Bacteroidetes and Proteobacteria. In comparison with young adults, in the >60 years age group, the relative abundance of Proteobacteria was higher (69 ± 32% vs 37 ± 18%) and lower in Firmicutes (11 ± 7% vs 33 ± 9%) and Bacteroidetes (13 ± 6% vs 17 ± 8%) (Fig. 2A). At the genus level, in the older adult group, the most abundant were *Bacteroides* (9 ± 4%), *Escherichia-Shigella* (69 ± 21%) *and Ezakiella* (9 ± 6%), while in young adults were *Escherichia-Shigella (37 ± 21%), Ezakiella (29 ± 18%)* and *Campylobacter (12 ± 9%)* (Fig. 2B).

### Richness and diversity of the gut microbiota

**Fig. 3:**
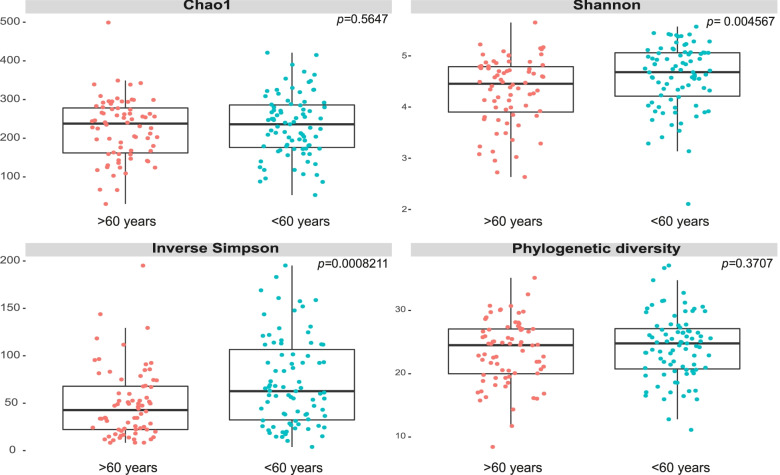
Analysis of alpha diversity in the age groups. Richness and diversity in the gut microbiota of older patients over 60 years old (pink) and patients under 60 years old (blue) in critical illness. Chao1, Shannon, Simpson’s reciprocal and phylogenetic diversity index. Wilcoxon range test was performed to analyze statistical significance between groups

**Fig. 4:**
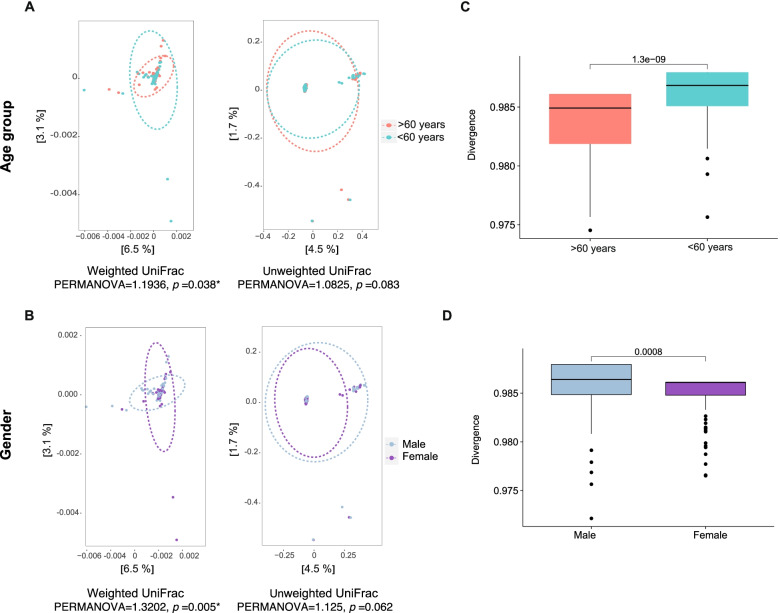
Structure of the microbial community. Principal coordinate analysis (PCoA) based on weighted and unweighted UniFrac distances by age and gender groups. The boxplot on the right quantifies the divergence of gut microbiota between groups of the variables represented

In the 155 samples, the number of ASVs ranged from 30 to 462. No differences were found in the Chao1 richness of gut microbiota among the age groups, but the diversity in the gut microbiota was lower in patients older than 60 years (Shannon index *p* = 0.004567, reciprocal Simpson index *p* = 0.00082; Wilcoxon rank test) (Fig. 3). Beta diversity assessed gut microbiota dissimilarity by weighted and unweighted UniFrac distance-based PCoA analysis, in weighted UniFrac, the results showed significant differences between the groups (PERMANOVA = 1.19, *p* = 0.038) with a less bacterial diversity in the group over 60 years old, thus, that age explained by 9.6% the variability of gut microbiota in the study (Fig. 4A). Similarly to age, the gender also significantly explained the variability of gut microbiota (PERMANOVA = 1.32, *p* = 0.005) (Fig. 4B). Confirming the previous results, the divergence index showed significantly less dissimilarity in the gut microbiota of adults older than 60 years (*p* < 0.001) of female gender (*p* = 0.0008) (Fig. 4C, D).

### Association of gut microbiota with variables

**Fig. 5:**
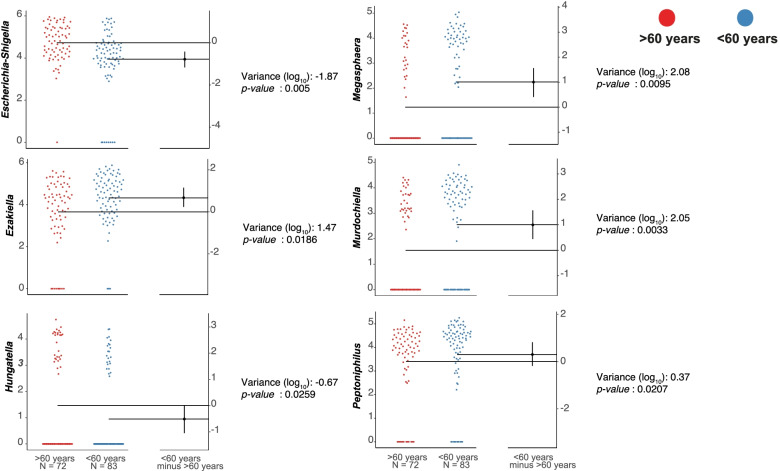
ASVs associated with the age group. Gardner-Altman estimation plots display effect size as the mean difference between age group (∆) and is shown on the right of the respective plots. ∆ is plotted as a black dot which indicates the resampled distribution of ∆, it is reported on a log scale and is referred to as observed in the patients over 60 years old group (*p* ≤ 0.01). The 95% confidence interval of ∆ is indicated by the ends of the vertical error bar

Through the mixed linear model, the demographic, biochemical, and clinical variables of the study were integrated with the most abundant ASVs (*n* = 251 with an average abundance>0.02%). A high specificity was identified, indicating that ASVs were strictly associated with each variable. Gender, discharge condition and BMI showed the highest number of associated ASVs. After adjusting the model (with the ASVs that showed a significant association (*p* < 0.05), the model was fitted to control the effects of other variables), a significant decrease in the *Ezakiella* was found in women compared to men, and for the age group, the variable of interest in this study, six bacterial groups were identified that were strongly associated with the age of the critical patients (Table 2). Specifically, adults under 60 years of age showed greater abundance of *Murdochiella, Megasphaera, Peptoniphilus* and *Ezakiella*, while in adults older than 60 years, the abundance of *Escherichia-Shigella* and *Hungatella* was greater (Fig. 5).

**Table 2:** Interaction^a^ between the most frequent ASVs (*p* < 0.05) and analyzed variables

Variable	Groups^**a**^	ASV_**S**_ associated	Top ASVs associated	***p***-value	Adjusted ***p***-value
Gender	**Male**/Female	16	*Lactobacillus*	0.0002	0.4902 (2.87)
			*Ezakiella*	0.0037	0.0186 (−1.12)
			*Oscillibacter*	0.0050	0.9653 (0.96)
			*Eisenbergiella*	0.0080	0.6948 (1.21)
			*Desulfovibrio*	0.0091	0.8548 (−0.16)
Discharge condition	**Dead**/Alive	11	*Ruminococcaceae UCG 004*	0.0014	0.3616 (1.45)
			*GCA 900066575*	0.0021	0.8945 (0.73)
			*CAG 352*	0.0109	0.8616 (0.43)
			*Brachybacterium*	0.0201	0.8327 (0.31)
			*Ruminococcus gauvreauii*	0.0244	0.9143 (0.33)
BMI	**Adequate**/Malnutrition	10	*Gallicola*	0.0002	0.3271 (1.62)
			*W5053*	0.0009	0.1710 (2.02)
			*Ezakiella*	0.0012	0.1376 (2.30)
			*Corynebacterium*	0.0048	0.258 (1.38)
			*Elusimicrobium*	0.0060	0.3721 (1.02)
ICU stay (days)	**High**/Medium	7	*Catenibacterium*	0.0084	0.517 (−1.00)
			*Candidatus.Soleaferrea*	0.0156	0.3882 (−1.24)
			*Prevotella 1*	0.0228	0.2848 (−0.98)
			*Ruminiclostridium 9*	0.0263	0.6419 (0.70)
			*Coriobacteriaceae UCG 003*	0.0278	0.3588 (−0.27)
Age group (years)	**>60** / <60	6	*Murdochiella*	0.0017	***0.0033*** *(2.05)*
			*Escherichia-Shigella*	0.0037	***0.005*** *(−1.87)*
			*Megasphaera*	0.0059	***0.0095*** *(2.08)*
			*Peptoniphilus*	0.0087	***0.0207*** *(0.37)*
			*Hungatella*	0.0112	***0.0259*** *(−0.67)*
			*Ezakiella*	0.0199	***0.0186*** *(1.47)*
Antibiotics	**Yes**/No	5	*Desulfovibrio*	0.0163	0.8548 (0.43)
			*Aggregatibacter*	0.0324	0.1946 (0.36)
			*Ruminococcaceae NK4A214*	0.0452	0.1229 (−0.12)
			*Filifactor*	0.0459	0.3059 (0.68)
			*Victivallis*	0.0463	0.6807 (0.89)

^a^Linear mixed model and adjustment by interactions. The reference group for the comparison is highlighted in bold. The most representative ASVs associated with each variable included in the model are shown; after adjusting for all factors, the last column shows the *p*-value and in parentheses the sense of the interaction

## Discussion

Under normal conditions, the microbiota and the components of the intestinal environment act harmoniously to maintain a symbiotic relationship, which benefits both the host and the bacteria themselves, and is achieved through a balance between intestinal integrity, inflammatory / anti-inflammatory response and bacterial diversity [24], conditions that may be compromised in older adults [25]. About 50% or more of ICU admissions correspond to older adults, patients in whom it is important to consider the comorbidities and fragility that accompany. It has been suggested that these patients tend to have an increased risk of complications and mortality [26].

In healthy individuals, Firmicutes and Bacteroidetes constitute at the phylum level 90% of gut microbiota [27]. Of interest is the finding in older adults, in which the abundance of Proteobacteria, associated with pathogenic bacteria, was higher than in young adults (69% vs 37%), suggesting a more pathogenic gut microbiota, which could be due to immunosenescence, process of immune dysfunction that occurs with age, a condition that could explain the overgrowth of opportunistic pathogens [28]. This is in line with what has been reported in critically ill patients, in whom severe physiological alterations and dysbiosis [29] turn the microbiome into a pathobiome [30], with loss of bacterial diversity and dominance of pathogenic microorganisms [24, 31, 32].

It is known that the aging process produces changes in the intestinal physiology (gastric hypochlorhydria, motility alterations, use of medications, among others) which can produce important modifications in the composition, diversity and functionality of the gut microbiota [33]. In older adults, with or without preexisting illnesses who are not in critical condition, changes in the diversity of gut microbiota have been described after the age of 70. Nevertheless, both a reduction diversity [34], as well as an increase in bacterial diversity, are reported. Although it is not clear if age is the cause or the effect [35]. In this study, upon admission of the patients to the ICU, the alpha diversity indices showed a loss of bacterial groups in older adults (*p* < 0.05) plus there is no changes in abundance compared to young adults. Regarding beta diversity, results showed that being a woman and older than 60 years old explains the variability of gut microbiota in 9.6% (*p* = 0.038), in addition to a less dissimilar microbiota. Indicating that this group of the Colombian population, in particular, is more vulnerable to the IM alterations and could represent an elevated risk of pathogen colonization.

Our findings are consistent with a study in a non-hospitalized Colombian population, in which the importance of taking age and sex into account as covariates in the analysis of gut microbiota is emphasized [36]. Given the conditions of the patients in our study, it becomes even more relevant to consider their effect on the evolution of the disease during the ICU stay, on the prognosis, the risk of complications and the mortality.

The results suggest a pattern of gut microbiota associated with the age of the patients in this study. In older adults an increase in *Escherichia/Shigella* and *Hungatella*, genera recognized as pathogens have been found. *Escherichia-Shigella*, recognized by their pathogenic and infectious potential, correspond to genera of Gram-negative bacteria, facultative anaerobes. *Shigella* has been associated to diarrheal infectious processes and *Escherichia* to infections of the urinary tract, gastrointestinal disorders, and pathological conditions [37]. While *Hungatella* is an anaerobic bacteria isolated from feces and blood; one of its species, the *hathewayi* has been identified in patients with bacteremia and liver abscess [38].

However, in young adults, was found a significant increase of the genus *Murdochiella*, Gram-positive bacteria. This bacterium has been isolated from patient wounds, producing significant amounts of lactic acid and moderate amounts of acetic, butyric and succinic acid [39].

Regarding the genera considered beneficial, due to their clinical importance, a decrease in *Megasphaera, Peptoniphilus* and *Ezakiella,* were also found in older adults. The first one, *Megasphaera*, obligate Gram-negative anaerobes belongs to the phylum of the Firmicutes and during *in vitro* studies, some of its species have shown a potential ability as membrane transporters, antibiotic resistance, producers of short chain fatty acids, vitamins and essential amino acids, among others. Although *in vivo* studies are required, the authors propose that this bacterium has a potential positive effect on host health [40] and is recognized as a producer of butyrate [41]. *Peptoniphilus*, an anaerobic Gram-positive is a butyrate producer, a short-chain fatty acid recognized for its anti-inflammatory effect, this genus is commensal in the human vagina and intestine [42]. Finally, *Ezakiella* is an anaerobic Gram-positive genus, which includes many commensal species as well as some human pathogens. It has been associated with the consumption of high complex carbohydrates diets in non-industrialized societies [43, 44].

Gut microbiota in older adults has been the object of several studies, among them the ELDERMET in Ireland, which followed individuals for years with the purpose of evaluating changes as their age increases. They have reported greater inter-individual variation in gut microbiota in those over 65 years of age and an important relationship with diet and living at home or being institutionalized [45]. Although the available evidence on the profiles of gut microbiota in critical adult patients has increased considerably over the last five years [2, 46–48], to date, there are no studies that describe in particular the gut microbiota of older adults admitted to the ICU. This study may be one of the first to report, after careful control of variables, the severe dysbiosis that older adults present during critical illness. The magnitude and relevance of these changes is still not well understood and new studies are required to describe the profiles of gut microbiota in older adults, not only upon admission to the ICU, but also until discharge from the hospital, and even up to 6 months after it.

Although both groups of patients were in critically ill, and the microbial community was altered by different factors, the findings of the study allow us to conclude that older adults are more vulnerable to severe and negative changes in gut microbiota. Age seems to be a determining factor towards a gut microbiota profile that suggests the presence of a pathobiome, which can increase the risk of complications, days of hospital stay and mortality. The impact of the aging process on alterations in the composition and function of gut microbiota will continue to be on the research agenda [49]. Likewise, strategies such as fecal microbiota transplantation, the contribution of prebiotics, probiotics or symbiotics in enteral nutrition formulas could help modulate the intestinal microbiota to avoid the loss of abundance and diversity, since these formulas are the main type of nutritional support for patients in the ICU.

However, more knowledge from observational work like this is needed on the composition of the intestinal microbiota in critically ill patients to better understand the clinical consequences of alterations in microbiota. Better comprehension on the mechanisms by which supplement therapies could restore microbiome homeostasis in order to apply them effectively and safely in clinical practice is also needed.

## Conclusion

Several factors impact on the composition of gut microbiota. After fitting a linear model, the age significantly explains the variability of the intestinal microbiota. Critically ill older patients have a lower bacterial diversity and a higher abundance of pathogens compared to young adults.

## Supplementary Information

**Additional file 1 Table S1**. Metadata of samples included in this meta-analysis.

## Data Availability

The raw fastq sequences generated from the 16S amplicon sequencing of fecal DNA are publicly available at the European Nucleotide Archive (ENA) via the project number PRJEB33360.
